# Assessment of Health-Related Quality of Life for Caregivers of Alzheimer's Disease Patients

**DOI:** 10.1155/2016/9213968

**Published:** 2016-12-19

**Authors:** Maria I. Andreakou, Angelos A. Papadopoulos, Demosthenes B. Panagiotakos, Dimitris Niakas

**Affiliations:** ^1^Faculty of Social Sciences, Hellenic Open University, Patras, Greece; ^2^Second Department of Internal Medicine, Medical School, Attikon University General Hospital, Athens, Greece; ^3^Department of Nutrition and Dietetics, Harokopion University, Athens, Greece; ^4^Medical School, National and Kapodistrian University of Athens, Greece

## Abstract

*Background.* Alzheimer's disease (AD) dementia is a chronic neurodegenerative disorder that results in total cognitive impairment and functional decline. Family members are the most usual caregivers worldwide, resulting in a subsequent degradation of their quality of life.* Methods.* During November 2013–March 2014 in Athens, Greece, 155 AD patients' family caregivers' Health-Related Quality of Life and existence of depressive symptomatology were assessed.* Results.* A strong negative correlation between the dimensions of HRQoL and the scores of the depression scale was revealed. AD patients' caregivers have a lower HRQoL almost in all dimensions compared to the Greek urban general population. The caregivers' social role, the existence of emotional problems, and their mental health status led to this result. Furthermore significantly important differences in caregivers' total HRQoL and depressive symptomatology were indicated in relation to their gender, hypertension existence, patient care frequency, cohabitation with the patient, disease aggravation, and economic status.* Conclusions.* Caring for relatives with AD strongly correlates with negative caregivers' HRQoL scores and adversely affects their depressive symptomatology. This negative correlation is enhanced in the later stages of the disease, in greater frequency of care, through living with a patient, in poor financial status, and with the existence of a chronic illness.

## 1. Introduction

Alzheimer's disease (AD), the most common form of dementia, is the term that describes the loss of mental abilities in a variety of areas of cognition, such as memory, speech, executive functions, and visuospatial skills. This loss is so severe that it leads to the impairment of everyday, professional, and social activities of the individual [1, 2]. Approximately 26.6 million people worldwide are living with AD. This number is expected to quadruple to more than 100 million by 2050 [3–5]. In 2010 the worldwide cost of dementia was 604 billion dollars. In Europe alone, overall expenditures related to dementia exceeded 170 billion € by 2006 [4].

Caregiving is by definition a very stressful task, especially when the patient faces a chronic, degenerative disease that presents several challenges such as AD [6]. Although the loss of recent memory is one of the early symptoms of the disease, the gradual loss of decision-making, orientation, and finally communication requires increased levels of supervision and personal care. At the final stages of the disease, patients may be completely dependent on their caregivers, even for basic daily activities [7] such as eating and bathing. One option for the provision of this level of care on a consistent basis is to provide it in an organized facility like a nursing home through “formal” caregivers, such as nurses and doctors. More often, however, care is provided by relatives (usually spouses and adult children), in an “informal” vis–à–vis unpaid fashion. These caregivers, without formal training, may end up both emotionally and physically exhausted [8, 9] due to the round-the-clock involvement with the patient. This is why they have often been called “the hidden victims” of AD [10, 11]. The caregiver's “burden” is a term coined to describe the accumulation of problems ranging from the stress involved in caretaking for AD patients to social isolation and financial problems that eventually could damage a caretaker's own professional and social life, physical and mental health, and financial prosperity [7, 9, 12–14].

In Greece, according to the European Alzheimer Organization (2012), there are approximately 202.000 dementia patients. It is estimated that ninety percent of these patients live at home and are cared for by a family member. There are no official data for dementia costs in Greece. By utilizing cost indicators from other developed countries, however, the annual cost of dementia in Greece may be as high as 3 billion euros. The role of caregivers in Greece, especially in relation to AD patients, has rarely been studied. The aim of this study is to estimate the physical heath and psychological well-being of relatives who care for AD patients, measuring the effects on the Health-Related Quality of Life (HRQoL) and the existence of depressive symptoms induced from the care.

## 2. Methods

This is an observational study that was designed in order to evaluate descriptive characteristics as well as associations between sociodemographic and emotional parameters with time devoted in care for AD patients'.

### 2.1. Sampling

Between November 2013 and March 2014, 180 relatives-caregivers of AD patients (58,1 ± 13,4 years old) from the greater Athens metropolitan area were approached to voluntarily enrol in the study. The response rate was 86,1%, resulting in 155 participants. Of the 180 caregivers approached, 25 declined to participate; 5 did not consent; 8 cared for patients suffering from other forms of dementia; and 8 did not find time to carry out the interview. The selected sample of 155 participants is considered adequate to evaluate multiple adjusted effect size measures equal to 0.14 at 5% significance level of two-sided hypotheses, achieving statistical power equal to 95% (sample size calculations were performed in G-Power v 3.0.10, Kiel, Germany).

There was no control group in this study.

Identifying the different caregivers occurred through the assistance of the more established AD and Dementia Associations in Athens greater area, such as Nestor Psychogeriatric Association, Athens Association of AD and other Dementia, Karelleio Standard Alzheimer Center, Iasis Amke Day Center, Dementia Special Clinic in Laiko University General Hospital, and Memory Disorder Clinic in Gennimatas General Hospital.

The inclusion criteria for caregivers were as follows: (a) age ≥ 18 years; (b) ability to communicate in Greek; and (c) providing written informed consent. Exclusion criteria were as follows: diagnosis of dementia other than AD. All participants were informed about the aims of the study and gave their consent to participate in the face-to-face interviews. The guided interviews were carried out at the associations' and public hospital facilities. In special circumstances concerning the patient's clinical condition, the interview was arranged to take place by telephone. The Ethics Committee of the Hellenic Open University gave approval for the study.

### 2.2. Measurements

A structured, close type questionnaire was used to retrieve sociodemographic and household information from the participants. The questionnaire included questions about age, sex, education, family status, self-reported medical history of hypertension, dyslipidemia, diabetes, coronary artery disease, heart failure, COPD, and arthritis, as well as questions about the relationship with the patient (spouse, child, brother, or others) and their living conditions. In particular, they were asked whether they live with the patient and asked about the number of other housemates and the size of the house as well as the housing ownership. The educational level of the participants was classified into three groups-levels (primary education, secondary education: up to high school or technical colleges, and higher education: university, masters, and doctorate degree). Moreover, participants were also asked to evaluate their financial status (report it as good, moderate, or bad) and report potential participation in patient's medical expenses. Finally, the stage of the disease as well as the overall time that the caregiver has been caring for their relative (in years, days a week, and hours per day), as well as potential attendance to caregivers' support programs, was also recorded.

Depressive symptomatology was assessed using a translated and validated version of the Zung Depression Rating Scale (ZDRS) [16, 17]. Worldwide the ZDRS is one of the most popular items used for self-rating measurement of depression. This self-reporting instrument was originally developed to estimate depression symptoms unbiased by administrators influence in the results. ZDRS consists of 20 elements that cover affective, psychological, and physical symptoms. The patient determines the frequency with which the symptom is experienced (i.e., a little = 1, some = 2, a good part of the time = 3, or most of the time = 4). Score of 80 is considered as maximum and score of 20 as minimum. The ZDRS scores fall into four ranges: normal range and mild, moderate, and severe depression. A subject with a ZDRS score of 70 or above is regarded as suggestive for severe depression, scores of 60–69 indicate moderate depression, while subject with a score of 50–59 is considered as mildly depressed and that with scores below 50 is considered normal [16–18].

Previous studies render ZDRS to be a valid and sensitive measure ideal as a research instrument for clinical severity in depressed patients and support its continued use [19]. Moreover, besides the sensitivity of the ZDRS, the adequacy of the scale [17, 19] was able to sufficiently differentiate four severity groups classified on the basis of the global rating.

Health-Related Quality of Life evaluates the impact of physical and mental disorders on the general well-being of a person [20]. Among the different questionnaires for assessing HRQoL, the Short Form 36 Health Survey (SF-36), developed by the Medical Outcome Study (MOS) for use in clinical practice and research, health policy evaluations, and general population surveys, is the most used one worldwide [21]. The SF-36 questionnaire, translated and validated in Greek [15], describes eight dimensions with scale score ranges from 0 to 100 (percent of maximum sum score, worst to best health state); it covers four physical health perceptions (physical functioning—PF, role limitations because of physical health problems—RP, bodily pain—BP, and general health—GH) and four mental health concepts (vitality—VT, social functioning—SF, role limitations because of personal or emotional problems—RE, and mental health perceptions—MH) [15, 22]. Twenty-eight items are in ordinal type following the Likert format (i.e., PF items: yes limited a lot recoded 1; yes limited a little recoded 2; no not limited at all recoded 3) seven items are in binary format (yes-no recoded 1-2), and one item, investigating the health changes over the past year, is not used for HRQoL evaluation. Both current and standard (4 weeks) recall versions are used. Successively these eight scales can be derived into two global measures, referred to as physical component summary (PCS) and as mental component summary (MCS) [15, 22, 23]. The MCS and PCS scores, which were designed to be independent and uncorrelated with each other, have been shown to have sufficient reliability and validity in indicating Health-Related Quality of Life [24].

### 2.3. Statistical Analysis

Continuous variables are presented as mean ± standard deviation and median (due to nonconvergence of the distributions with the normal distribution). Categorical variables are presented as frequencies. Correlations between categorical variables were tested by calculation of Pearson's chi-squared test. Comparisons between continuous variables following normal distribution or with general population's SF-36 scores were performed by measuring the control Student's* t*-test. In the case of continuous variables not normally distributed control cases were evaluated using the nonparametric *U* test criterion which was proposed by Mann and Whitney. Correlations between continuous variables were assessed using linear correlation coefficient Spearman rho. The normality of variables was tested using PP charts. For further investigation of the physical and mental health along with depressive symptomatology of caregivers of AD patients, versus various sociodemographic factors, chronic diseases, stage of disease progression, lineage relationship, cohabitation with the patient, and weekly care, linear regression models were also produced. The associations between, age, gender, level of education, chronic diseases (hypertension, dyslipidemia, diabetes, coronary artery disease, heart failure, COPD, and arthritis), degree of kinship, stage of illness, cohabitation, days a week of care, financial status (dependent variables), and ZDRS score, physical component summary score (PCS), and mental component summary (MCS) score (independent variables) were tested through the multiple linear regression analysis. The results from the regression models are presented as* b*-coefficients and standard error of the coefficient. All statistical analyses were performed using the SPSS version 18.0 (SPSS PREDICTA Hellas).

## 3. Results

### 3.1. Characteristics of the Participants

Sociodemographic and financial characteristics of participants are listed in Table 1. The majority of caregivers were adult children of the patient (48,4%), 74,8% were married, and 48,4% had secondary education. The patients' medical expenses were covered by their own or the spouse's retirement pension in 72,3%, while for the 23,9% of the patients the caregivers also covered part or all of the medical expenses, although 67,1% stated that their economic status is moderate.

### 3.2. Medical and Habitation Characteristics of the Participants and Level of Care Provided

Self-reported health status of the participants, living conditions, time engaged in the care, and stage of illness of the patients are outlined in Table 2. The reported history of hypertension in a rate of 32,9% and dyslipidemia (18,1% of the sample) were the most important comorbidities among the chronic diseases surveyed.

Moreover, the majority of the patients (85 out of the 155, percentage of 54,8%) were clinically diagnosed at the second stage of AD disease. The caregivers provided assistance for 4,6 ± 3,7 years, with an average of 7,1 ± 5,2 days of weekly care and 10,1 ± 7,4 hours of daily care. While 69,7% of the caregivers resided with the demented patient, only 27,7% of the whole group had ever received any formal education in caregiving, such as informational seminars, psychoeducational, skills-training, and therapeutic counselling interventions to help offset their burden. Due to the small percentage of caregivers receiving help there was no statistically significant association between the participation in such courses and depressive symptomatology and quality of life of the participants.

### 3.3. Depressing Symptomatology and Quality of Life of the Participants

The distribution of the SF-36 and ZDRS scores of caregivers of the study is presented in Table 3. Higher scores are found in physical functioning (PF 80 ± 22,5), while the lowest ones are in the emotional role because of the caregivers' emotional problems (RE 44,7 ± 44). An inverse linear association was observed between depressive symptomatology and the eight dimensions, the four physical health perceptions, and the four mental health concepts of the quality of life of the participants. As a consequence both the two component summary scales were inversely associated with the ZDRS scores.

### 3.4. Depressive Symptomatology, Quality of Life and Levels of Care, and Demographic, Clinical, and Habitation Characteristics of the Participants

The associations from the multiple linear regression analysis performed between the ZDRS, PCS, and MCS scores (independent variables) and gender, age, years of school, financial level, chronic diseases, living conditions, progression of illness, weekly care, and family relationship (dependent values) are shown in Table 4. It seems that gender (women versus men) was highly associated with the physical and mental quality of life along with depressive symptomatology of the women caregivers. In addition the presence of hypertension, increased frequency of care (more hours per week), cohabitation with the demented relative, and poor financial state of the caregivers as well as the progression of AD disease aggravated the caregivers' HRQoL and their depressive symptoms.

### 3.5. Quality of Life of the Participants in Comparison to the General Population

Comparisons of SF-36 dimensions between the sample of the study's caregivers and an age-sex matched sample from the dataset of the Greek general population retrieved from Pappa et al. [15] are shown in Table 5. It seems that the family caregivers of AD patients have a lower HRQoL in all dimensions compared to the Greek urban general population, except the scores in physical functioning in which the caregivers have higher scores. The statistically significant association results in general health and the mental health concepts: social functioning and emotional role limitations (role limitations because of personal or emotional problems) and mental health perceptions.

## 4. Discussion

Comparing the SF-36 scores of the population of the present study to those of the Greek urban general population as they were presented at another survey that was also conducted in Athens [15], family caregivers of AD patients have a lower HRQoL in almost all dimensions with exception of the subjective assessment of the level of physical functioning. The greatest burden seemed to come from the existence of emotional problems. Also, in comparison to the general population, a much higher burden, for the caregivers, appeared to result from their social role and be due to their mental health status. Moreover, our results appear to follow results of studies in other countries [2, 8, 12, 14, 25–34]. Female caregivers, who were also the majority of caregivers, appear to face a greater adverse impact in both quality of life and depression measurements than their male counterparts [8, 9, 12, 14, 31–36]. Analysis of cohabitation relationship also remains consistent with literature, as caregivers who lived with the care recipient seemed to experience more severe symptoms of depression and had poorer mental dimension of quality of life, compared to caregivers living separately [8, 30, 37]. Furthermore, according to the survey results, the self-reported economic status was found to affect both quality of life and levels of depression [9, 14, 31]. The multiple linear regression highlighted the inverse relationship of the transition from best to worst economic situation and the concurrent reduction of physical health and increased depression. The involvement of caregivers in the patients' medical costs was not correlated, however, as was expected, despite the large financial cost involved in the treatment of the demented and the challenging fiscal conditions in Greece. We also found a strong relation between HRQoL and the stage of disease progression: as if it is deteriorating, the mental health and psychology of the caregiver are affected negatively [6, 8, 9, 14]. In addition, the more the time a caregiver spends caring for patients the lower the caregivers' QoL score [2, 8, 14, 30]. Other studies have linked caregiving induced stress to cardiovascular complications especially hypertension [12, 27]. In our study, it is evident that caregivers with hypertension have worse physical quality of life and experience stronger symptoms of depression, compared to those without hypertension. The lack of readily available demographics for the Greek population of AD caregivers does not, however, allow us to make broader statements based on our findings.

The link between AD caregiving and higher rates of physical and mental disorders that can cause deterioration of quality of life has been well established in the literature [22, 31, 33]. Social services need to aim at developing more targeted interventions to support caregivers since the latter run the risk of higher depression rates and physical vulnerability, especially if they receive neither formal nor informal support [5, 12, 14, 35]. Support can range from functional support (helping with the demanding daily living needs and housework) to emotional support and/or informational support, which includes sharing knowledge from health professionals and individuals that have experienced similar situations [29, 37–39]. The positive relationship between receiving formal or informal support and a caregiver's psychological well-being [26] and overall QoL may lead to better quality of care and reduction of the possibility of institutionalization of the AD patient [9]. Exposing the caregiver to multicomponent interventions [5, 13, 40] can increase knowledge (i.e., attending an information session or a skills building program) and reduce depression levels [5, 36]. Psychosocial interventions such as behaviour management therapy, psychological ad hoc counselling (both family and individual), participating in a specialized support group, and stress management techniques can improve outcomes for caregivers [5, 12, 29, 37–39] and in so doing also improve the quality of life for patients. The small number of participants in our study who had received support of some fashion (only 43 of the 155 participants) was, however, a limitation and possibly explains the lack of statistical significance in the association between the participation in such courses, the depressive symptomatology, and quality of life of the caregivers, despite all the previous findings in the literature.

As our findings indicate, women were more adversely affected compared to men in terms of both QoL and depression. This can mainly be explained by the fact that women take up more exhausting activities such as patient's personal hygiene and managing household chores [8, 30, 32] while balancing other life responsibilities, including child rearing, career, and relationships. Moreover, our data indicate that the mental quality of life and the depressive consequences of the informal caregivers are both closely correlated with the severity of AD. An AD patient who is in the later stage needs more assistance even for daily mundane routines in comparison to patients in earlier stages. This in turn may increase the burden on the caregiver and increase his/her levels of physical and psychological exhaustion [2, 6, 8, 9, 28]. Moreover, as other studies have shown, AD patients' caregivers often relate their emotional problems to physical illness such as cardiovascular diseases, reduced wound healing capacity, and reduced overall body immunity [27]. We also found in this study strong correlations between hypertension, depression, and physical quality of life.

### 4.1. Limitations and Strengths

The present study bears some limitations. The basic limitation is that it cannot draw any definitive conclusions about cause and effect results and, therefore, only informed assumptions can be made for the underlying relationships that affect AD patients' caregivers' physical and mental health. Comparisons were made only to data from epidemiological reference population, since there is no registered representative sample for the population of our study in Greece. However, comparison to a control group is required to fully assess the magnitude of the consequences brought upon the AD caregiver. In addition, the survey was conducted in only one region of the country, which, as representative as it might be since half the Greek population resides in the capital, does not fully cover the entire societal spectrum. Cross section investigation of all regions should take place. Moreover, the fact that participants did not enter the caring role at identical intervals, as well as the fact that they did not face similar care challenges at the same time, may also have affected the scores recorded. The interview of the same caregivers at different times as part of an overall cohort study could give a clearer picture of the quality of life and depressive symptoms experienced.

However this work also has some merits; the study is of the few of its kind in the Greek setting and could therefore serve as the basis for further funding and research as well as public health policies in relation to AD caregiving. Important and necessary functional, emotional, and financial support could be provided to assist relatives who serve as informal caregivers cope with the burden of caring and in this way increase their quality of life and overall economic well-being.

## 5. Conclusions

In conclusion, this study has shown that taking care of a family member with AD negatively affects HRQoL and depressive symptomatology. The presented findings provide strong support for exploiting the possibility of offering functional, emotional, and financial support to the caregiver. Local dementia associations could provide information, emotional support, practical advice, support groups, and training programs, assuming that greater financial support for that cause becomes available either through the state or through other fund raising processes. Under the prism of the current financial crisis that Greece faces, a National Action Plan could present a roadmap of tackling the medical, social, and financial impact of dementia in our country, assist in the overall coordination of both health and social services among a number of currently fragmented structures, and initiate a national research policy. Finally, the development and implementation of such a National Action Plan on AD disease are of the utmost importance not only in terms of supporting patients and their caregivers but also in terms of achieving long-term rationalization of state resources available for the disease.

## Figures and Tables

**Table 1:** Sociodemographic characteristics and financial status of the participants (*n *= 155).

Age (in years)	58,1 ± 13,4
Main carer (yes), %	95,5%
Type of relationship	
Spouse, %	37,4%
Child, %	48,4%
Siblings, %	2,6%
Other relatives (in-laws, cousins, friends), %	11,6%
Partner, %	0,6%
Family status	
Single, %	11,6%
Married, %	74,8%
Divorced, %	11,0%
Widowed, %	1,9%
Educational status	
Primary education, %	14,2%
Secondary education, %	48,4%
Higher education, %	37,4%
Economic status	
Economic participation in care (yes), %	23,9%
Economic status stated to be good, %	9,0%
Economic status stated to be moderate, %	67,1%
Economic status stated to be bad, %	23,9%

**Table 2:** Self-reported health status of the caregivers, living conditions, and time engaged in the care.

History of hypertension, %	32,9%
History of dyslipidemia, %	18,1%
History of diabetes, %	8,4%
History of coronary artery disease, %	4,5%
History of heart failure, %	3,9%
History of COPD, %	3,2%
History of arthritis, %	13,5%
First stage of AD (patient), %	22,6%
Second stage of AD (patient), %	54,8%
Third stage of AD (patient), %	22,6%
Living with patient (yes), %	69,7%
Years of care	4,6 ± 3,7
Days/week of care	7,1 ± 5,2
Hours/day of care	10,1 ± 7,4
Participation in any course for caregivers (yes), %	27,7%

AD = Alzheimer's disease. COPD = chronic obstructive pulmonary disease.

**Table 3:** Depression (ZDRS) and quality of life (SF-36) scores of the caregivers and correlation coefficients between ZDRS and SF-36.

	Mean ± SD	*r*, *p *value
ZDRS score (20–80)	42.9 ± 10.7	
SF-36 dimensions score (0–100)		
*Physical functioning*	80 ± 22.5	−0.434, (0.0001)
*Role limitations: physical*	65.5 ± 41.5	−0.462, (0.0001)
*Bodily pain*	68.7 ± 30	−0.422, (0.0001)
*General health*	64.7 ± 22	−0.666, (0.0001)
*Vitality*	57.8 ± 23	−0.761, (0.0001)
*Social functioning*	57.4 ± 33.7	−0.598, (0.0001)
*Role limitations: emotional*	44.7 ± 44	−0.576, (0.0001)
*Mental health*	52.7 ± 23.6	−0.462, (0.0001)
*Physical component summary*	50.6 ± 10	−0.342, (0.0001)
*Mental component summary*	37 ± 14	−0.774, (0.0001)

ZDRS = Zung Depression Rating Scale.

**Table 4:** Results from multiple linear regression models that evaluated sociodemographics, financial status, chronic diseases, habitation conditions, aggravation of illness, kinship, and weekly care.

Factor(s):	ZUNG score	PCS	MCS
	*b* ± SE, *p*	*b* ± SE, *p*	*b* ± SE, *p*
Age (in years)	−0,115 ± 0,078, (0.145)	−0.073 ± 0.081, (0.369)	0.171 ± 0.113, (0.132)
Men versus women	4,782 ± 1,815, (0.009)	−3.215 ± 1.864, (0.087)	−5.489 ± 2.609, (0.037)
Primary versus middle/higher education	0.372 ± 1.224, (0.761)	0.502 ± 1.263, (0.691)	−0.212 ± 1.768, (0.905)
Normotensive versus hypertensive	5.494 ± 2.038, (0.008)	−4.442 ± 2.095, (0.036)	−3.547 ± 2.932, (0.228)
Normal versus dyslipidemic	1.068 ± 2.028, (0.599)	0.477 ± 2.082, (0.819)	−1.403 ± 2.914, (0.631)
Nondiabetic versus diabetic	1.569 ± 2.979, (0.599)	−1.467 ± 3.056, (0.632)	1.368 ± 4.278, (0.750)
Coronary artery disease (Y/N)	−0.012 ± 0.006, (0.045)	−4.4 ± 3.863, (0.257)	−6.693 ± 5.407, (0.218)
Progression of AD (Y/N)	2.650 ± 1.138, (0.021)	0.465 ± 1.167, (0.691)	−3.103 ± 1.634, (0.060)
Cohabitation with patient (Y/N)	−7.950 ± 1.955, (<0.001)	2.128 ± 2.01, (0.292)	8.692 ± 2.814, (0.002)
Weekly care (0–7 days)	−0.079 ± 0,148, (0.592)	0.254 ± 0.152, (0.095)	−2.249 ± 0.212, (0.242)
Higher versus middle and low income	3.272 ± 1.517, (0.033)	−2,71 ± 1.568, (0.086)	−3.257 ± 2.195, (0.140)

AD = Alzheimer's disease, PCS = physical component summary, and MCS = mental component summary.

**Table 5:** Comparisons of SF-36 dimensions between study's sample and an age-sex matched sample from the general population (retrieved from Pappa et al. [15]).

	General population mean ± SD	Caregivers mean ± SD	*p *value
SF-36 dimensions score(s) (0–100)			
*Physical functioning*	74.8 ± 32	80 ± 22.5	0.168
*Role limitations: physical*	71.6 ± 46	65.5 ± 41.5	0.062
*Bodily pain*	70.5 ± 31	68.7 ± 30	0.455
*General health*	59 ± 24	64.7 ± 22	0.040
*Vitality*	61 ± 22.8	57.8 ± 23	0.123
*Social functioning*	78 ± 30.4	57.4 ± 33.7	<0.001
*Role limitations: emotional*	83 ± 72	44.7 ± 44	<0.001
*Mental health*	63.6 ± 20.6	52.7 ± 23.6	<0.001
